# Plasma Atrial Natriuretic Peptide Concentrations and Associated Factors in Captive Dolphins: Potential Implications for Cardiovascular Assessment

**DOI:** 10.3390/ani16081151

**Published:** 2026-04-10

**Authors:** Megumi Yamamoto, Kyogo Hagino, Chika Shirakata, Takaomi Ito, Keiichi Ueda, Mariko Omata, Akiko Uemura, Ryou Tanaka

**Affiliations:** 1 Graduate School of Animal Husbandry, Obihiro University of Agriculture and Veterinary Medicine, Obihiro 080-8555, Hokkaido, Japan; ymegumi@obihiro.ac.jp; 2 Noboribetsu Marine Park Nixe, Noboribetsu 059-0492, Hokkaido, Japan; 3 Enoshima Aquarium, Fujisawa 251-0035, Kanagawa, Japan; 4 Osaka Aquarium Kaiyukan, Minato-ku 565-0826, Osaka, Japan; 5 Okinawa Churashima Foundation, Motobu 905-0206, Okinawa, Japan; 6 Department of Clinical Veterinary Medicine, Obihiro University of Agriculture and Veterinary Medicine, Obihiro 080-8555, Hokkaido, Japan; 7 Department of Veterinary Surgery, Faculty of Agriculture, Tokyo University of Agriculture and Technology, 3-5-8 Saiwai-cho, Fuchu 183-8509, Tokyo, Japan

**Keywords:** captive dolphin, cetacean, atrial natriuretic peptide (ANP), cardiovascular disease, echocardiography

## Abstract

This exploratory study evaluated plasma atrial natriuretic peptide (ANP) concentrations in four species of captive cetaceans and their associations with physiological and environmental factors, including husbandry conditions, diet, and management practices. Twenty-six individuals were voluntarily sampled, and blood samples were analyzed using a human-based chemiluminescent immunoassay. Transthoracic echocardiography was also attempted in several individuals but was technically challenging due to interference from the lung tissue and the sternum. The mean plasma ANP concentration in clinically healthy young animals was 44.12 ± 14.62 pg/mL, with no significant differences observed according to age, sex, species, or the presence of mild chronic disease. ANP was detectable across all species using human reagents. In addition, brain natriuretic peptide (BNP), a commonly used cardiac biomarker in humans and other animals, was evaluated for comparison; however, BNP concentrations remained below the detection threshold, possibly reflecting species-specific differences. Importantly, ANP concentrations were not evaluated in animals with clinically significant disease, and therefore the relevance of these findings to diseased populations remains unknown. These results demonstrate that plasma ANP can be measured using existing clinical assays and provide preliminary baseline data for multiple cetacean species. Overall, ANP may offer preliminary insights into physiological variation in managed cetaceans; however, its utility as a biomarker for cardiovascular or systemic health assessment remains to be determined.

## Simple Summary

Health monitoring of captive dolphins is essential for the early detection of age-related diseases. Diagnostic imaging procedures may require specialized equipment or trained handling, but are commonly performed in cetaceans using voluntary medical training without anesthesia. This study investigated whether atrial natriuretic peptide (ANP), a cardiac hormone measured in blood, could serve as a minimally invasive indicator of cardiovascular stress. Blood samples were collected using voluntary behavioral training in multiple dolphin species. The results suggest the potential feasibility of ANP as a biomarker, which may improve daily welfare management in aquaria.

## 1. Introduction

Cardiovascular disease is increasingly recognized in captive marine mammals, including cetaceans; however, diagnostic challenges persist due to their unique anatomy and physiology. Transthoracic echocardiography has been applied in cetaceans using trained positioning techniques; however, image acquisition can be limited by anatomical factors such as lung interference and the sternum [1], as well as difficulties in probe positioning [2,3]. Moreover, echocardiographic imaging requires a high level of husbandry training and specialized equipment [2,3,4], which may be limited in some facilities depending on equipment availability and training level. These constraints hinder routine cardiovascular assessment and the early detection of subclinical heart disease in captive cetacean populations. As a result, there is a need for complementary, minimally invasive approaches that can be more readily implemented across a range of management settings.

Atrial natriuretic peptide (ANP) is a cardiac hormone primarily produced by atrial myocytes in response to volume and pressure overload [5,6,7,8,9]. In humans and dogs, plasma ANP levels correlate with atrial stretch and are clinically used as biomarkers of cardiac stress and heart failure [5,8,9,10,11,12]. However, ANP concentrations are also known to be affected by non-cardiac factors such as age, systemic inflammation, and endocrine disease [13,14]. In addition to its role as a cardiac biomarker, ANP is involved in the regulation of fluid balance, vascular tone, and neurohormonal activity, reflecting broader aspects of cardiovascular homeostasis [5,6,9]. Recent studies suggest that the mature ANP sequence is highly conserved among mammals, with high sequence homology between humans and several cetacean species [8,15]. This molecular similarity enables the use of commercially available human assay kits in dolphins, as demonstrated in bottlenose dolphins (*Tursiops truncatus*) [15]. Despite this, limited data exist regarding normal ANP concentrations and the effects of age, sex, species, and underlying conditions in cetaceans. Furthermore, the interpretation of ANP concentrations in cetaceans may be influenced by species-specific physiological adaptations, including those associated with diving behavior and aquatic life, which could affect cardiovascular regulation.

This study aimed to evaluate plasma ANP concentrations in four captive cetacean species: bottlenose dolphins, Pacific white-sided dolphins (*Lagenorhynchus obliquidens*), Indo-Pacific bottlenose dolphins (*T. aduncus*), and one hybrid dolphin (*T. truncatus* × *T. aduncus*). Using voluntary blood sampling and a human-based chemiluminescent assay, we examined the effects of physiological and environmental variables such as facility conditions, husbandry practices, and feeding status. These environmental factors were not strictly controlled, although several variables were descriptively evaluated. Additionally, we assessed the feasibility of transthoracic echocardiography and measured plasma brain natriuretic peptide (BNP) concentrations as a complementary cardiac biomarker [16]. BNP is a well-established cardiac biomarker in humans and other animals and was included for comparison with ANP [5,9,16]. Given the limited availability of validated biomarkers in cetaceans, a comparative approach using multiple cardiac-related peptides may provide additional context for interpreting physiological variation. In addition, the identification of practical and minimally invasive biomarkers may help complement existing diagnostic approaches and support routine health monitoring in facilities with varying levels of resources and training. Establishing a reliable, minimally invasive method for assessing cardiac health in cetaceans is critical for improving long-term welfare and disease management [17,18,19,20]. However, given the current lack of disease-specific validation, such approaches should be considered preliminary and require further investigation before clinical application. This study provides foundational data that may help facilitate routine cardiovascular monitoring and contribute to broader discussions on marine mammal health surveillance in zoological institutions.

## 2. Materials and Methods

### 2.1. Transthoracic Echocardiography

This section is included to evaluate the feasibility and limitations of transthoracic echocardiography in these animals, providing context for the use of blood-based biomarkers as an alternative or complementary approach.

#### 2.1.1. Animals, Examiners, and Equipment

Transthoracic echocardiography was performed in one female bottlenose dolphin (12.2 years old, 167.5 kg; Enoshima Aquarium, Fujisawa, Japan) and one male Pacific white-sided dolphin (estimated 16 years old, 117 kg; Noboribetsu Marine Park Nixe, Noboribetsu, Japan).

Examinations were performed using a veterinary ultrasound imaging device (MyLab^TM^ One VET, Esaote, Genova, Italy) equipped with a convex probe (SC3421, Esaote, Genova, Italy). The operating frequency was not specified, as it varied depending on imaging conditions and was adjusted as needed during the examination.

#### 2.1.2. Measurement Method at the Enoshima Aquarium

The animal was gently restrained on a stretcher in the shallow area of the pool. It was held in a lateral recumbency by its care staff, with the blowhole always positioned above the surface of the water. The examiners approached from the poolside and positioned the probe on the submerged lateral thorax, targeting the thoracic midline area.

#### 2.1.3. Measurement Method at the Noboribetsu Marine Park Nixe

The animal was trained to maintain a dorsal recumbent position in the water. During the examination, breathing was controlled through trainer cues rather than occurring freely. This approach allowed stable positioning during image acquisition.

### 2.2. Plasma ANP Concentrations

#### 2.2.1. Animals Included in ANP Analysis

This study included 26 dolphins: 13 bottlenose dolphins (2 males, 11 females; estimated 8.7–44.7 years old), 10 Pacific white-sided dolphins (7 males, 3 females; estimated 14–47.7 years old), 2 Indo-Pacific bottlenose dolphins (1 male, 1 female; estimated 51–54 years old), and 1 bottlenose dolphin/Indo-Pacific bottlenose dolphin hybrid (female; estimated 35 years old). These dolphins were bred in the Enoshima Aquarium, the Okinawa Churaumi Aquarium, the Kaiyukan, and the Noboribetsu Marine Park Nixe. The study included healthy dolphins as well as those presenting with renal dysfunction, hepatic dysfunction, and chronic inflammation. Animals were classified as clinically healthy based on routine physical examinations, standard hematological and biochemical analyses, and other diagnostic evaluations routinely performed at each facility; however, no standardized examinations were performed as part of this study. Basic health conditions were reported by the facilities, and no general examinations were performed in this study. Details of the dolphins are shown in Table 1.

#### 2.2.2. Measurements of Plasma ANP

Blood samples were collected voluntarily from the ventral fluke vein of each dolphin during routine fasted husbandry training sessions. Samples were immediately transferred into EDTA-2Na tubes containing aprotinin, then centrifuged (3000 rpm, 10 min). Plasma was separated, aliquoted, and immediately frozen at −20 °C until analysis. Plasma samples were analyzed using a chemiluminescent enzyme immunoassay (CLEIA) with a commercially available assay system (Fujifilm VET Systems, Tokyo, Japan) according to the manufacturer’s instructions. Environmental and procedural factors, such as differences in specimen handling and potential stress-related responses during sampling, were not strictly controlled, as samples were collected under routine husbandry conditions.

#### 2.2.3. Data and Statistical Analysis

The population was divided into two groups based on age, sex, species, and health condition. Differences between the groups were evaluated using the Mann–Whitney U test, with a significance level of *p* < 0.05. The groups used for the statistical analysis are shown in Table 2. Based on previous reports on the longevity of captive bottlenose dolphins [21], dolphins aged 30 years or younger were operationally defined as the young healthy group, and those over 30 years were defined as the elderly group. For the purposes of this study, the term “young” refers to clinically healthy adult animals and does not indicate juvenile or pre-pubertal individuals. In addition, five dolphins with chronic conditions including hepatic dysfunction, renal dysfunction, and/or chronic inflammation were defined as the diseased group to serve as a comparison for the young healthy group (aged 30 years or younger). In addition to these three groups, the male group, female group, bottlenose dolphin group, and Pacific white-sided dolphin group were defined from dolphins not belonging to the diseased group to evaluate sex and species differences. The two Indo-Pacific bottlenose dolphins were included in the elderly group, and the one hybrid dolphin was included in the diseased group.

Spearman’s rank correlation coefficient was calculated between plasma ANP concentrations and estimated ages for 21 dolphins from the combined the young healthy and elderly groups. Statistical analysis was performed using GraphPad Prism 8.2.1.

### 2.3. Plasma BNP Concentration

#### 2.3.1. Animals Included in BNP Analysis

This study included 10 captive dolphins: 8 female bottlenose dolphins (estimated 8.7–44.7 years old) and 2 male Pacific white-sided dolphins (estimated 26.7–47.7 years old). These dolphins were housed in the Enoshima Aquarium. Plasma ANP and BNP concentrations were measured from the same blood samples.

#### 2.3.2. Measurements of Plasma BNP

Plasma BNP concentrations were measured using samples collected and processed as described for ANP, except that blood was collected into tubes containing EDTA-2Na. Plasma BNP concentrations were measured using a chemiluminescent enzyme immunoassay (CLEIA) by a commercial laboratory (SRL Inc., Tokyo, Japan) according to the manufacturer’s standard protocols.

## 3. Results

### 3.1. Feasibility and Limitations of Transthoracic Echocardiography

Adequate echocardiographic images for cardiac assessment could not be obtained at either facility. Image quality was insufficient to allow reliable detection of cardiac abnormalities or disease. These findings highlight the technical limitations of echocardiography and provide context for exploring alternative biomarkers such as ANP.

#### 3.1.1. Findings at the Enoshima Aquarium

**Figure 1:**
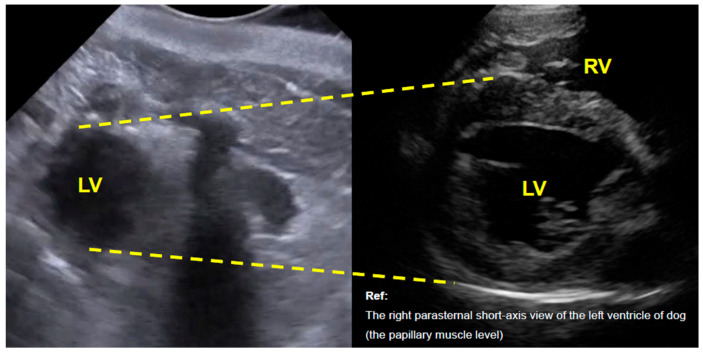
Transthoracic echocardiographic image of a bottlenose dolphin (*Tursiops truncatus*) in a right parasternal short-axis view of the left ventricle. The **right panel** shows a comparable cross-sectional image from a clinically healthy dog obtained during routine examination with owner consent. LV, left ventricle; RV, right ventricle.

In right lateral recumbency, with the reference marker oriented dorsally, a cross-sectional image corresponding to a right parasternal short-axis view of the left ventricle (distal to the papillary muscle level) was obtained (Figure 1). However, imaging was limited to this markedly apical region, precluding visualization of the right ventricle and assessment of left ventricular systolic function.

**Figure 2:**
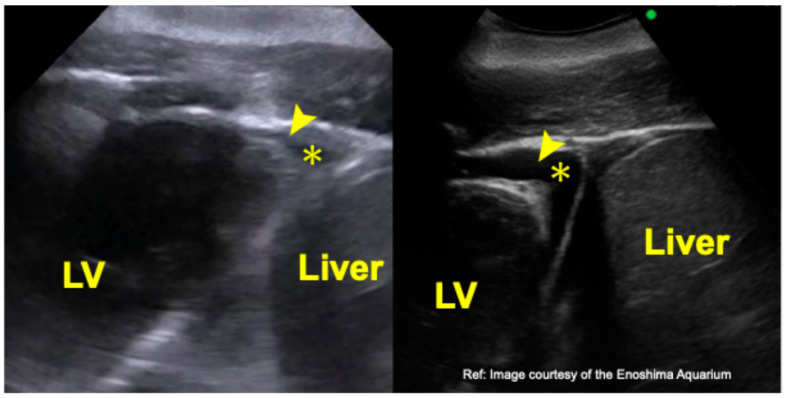
Transthoracic echocardiographic image of a bottlenose dolphin (*Tursiops truncatus*) obtained from the liver-left ventricular apical window. The **right panel** shows a comparable image provided by the Enoshima Aquarium (archival case). LV, left ventricle; arrowhead, left ventricular apex; *, physiological pericardial effusion.

With the same positioning and the reference marker oriented cranially, a cross-sectional image extending from the liver to the left ventricular apex was visualized (Figure 2). As in the previous image, the view was restricted to the apical region, preventing evaluation of the ventricular chambers and myocardial walls.

**Figure 3:**
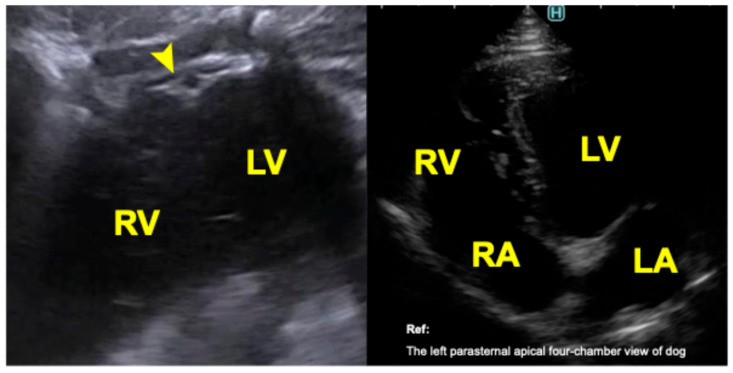
Transthoracic echocardiographic image of a bottlenose dolphin (*Tursiops truncatus*) obtained from the left parasternal apical four-chamber view. The **right panel** shows a comparable cross-sectional image from a clinically healthy dog obtained during routine examination with owner consent. LV, left ventricle; RV, right ventricle; LA, left atrium; RA, right atrium; arrowhead, cardiac apex.

In left lateral recumbency, with the reference marker oriented dorsally, an image partially corresponding to an apical four-chamber view was obtained from a left parasternal approach (Figure 3). This view included portions of both ventricles but did not allow visualization of the atria or cardiac valves.

#### 3.1.2. Findings at the Noboribetsu Marine Park Nixe

**Figure 4:**
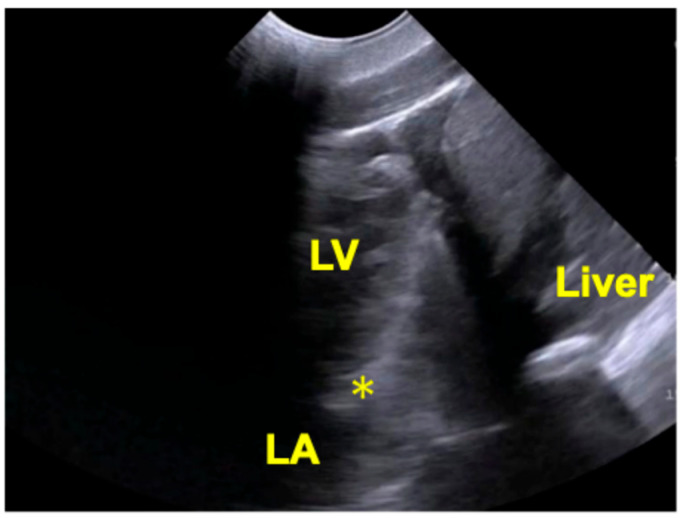
Transthoracic echocardiographic image of a Pacific white-sided dolphin (*L. obliquidens*) obtained from the liver-left ventricular apical window. LV, left ventricle; LA, left atrium; *, mitral valve-like structure.

In dorsal recumbency, with the animal’s head positioned to the examiner’s left, the transducer was placed slightly left of the midline with the reference marker oriented caudally. This approach allowed visualization of a longitudinal section extending from the liver to the cardiac apex, reaching a depth corresponding to the left atrial level (Figure 4).

However, visualization of the mitral valve was indistinct. Although a valve-like structure was identified and evaluated using pulsed-wave Doppler, no definitive flow signals were detected. When the transducer was angled further laterally, the imaging field shifted toward the left heart, limiting visualization of the right heart and the cardiac base.

### 3.2. Cardiac Biomarker

#### 3.2.1. Plasma ANP Concentration

**Figure 5:**
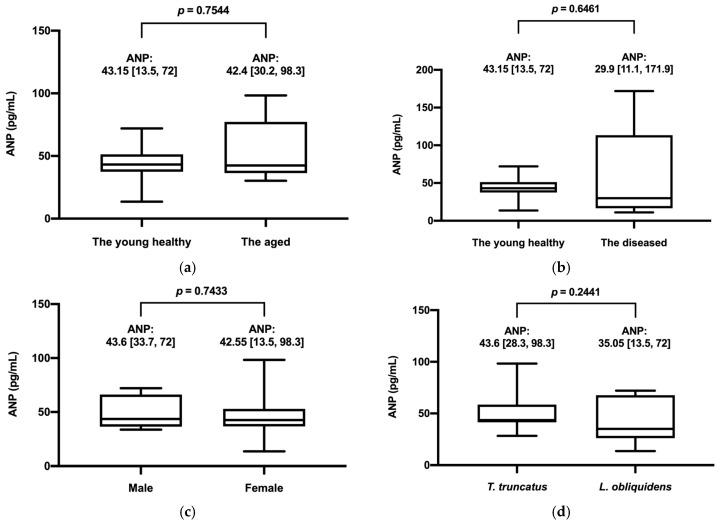
Comparison of plasma atrial natriuretic peptide (ANP) concentrations by age, disease status, sex, and species. (**a**) Young healthy vs. aged groups; (**b**) Young healthy vs. diseased groups; (**c**) Male vs. female groups; (**d**) Bottlenose dolphins (*Tursiops truncatus*) vs. Pacific white-sided dolphins (*Lagenorhynchus obliquidens*). Data are presented as mean [range]. No significant differences were observed between groups (*p* > 0.05).

In the 12 dolphins in the young healthy group, the mean plasma ANP concentration was 44.12 ± 14.62 pg/mL (mean ± SD). Group comparisons are shown in Figure 5. No significant differences were observed between the young healthy group and the aged group (*p* = 0.7544) or between the young healthy and diseased groups (*p* = 0.6461), indicating no clear associations with age or disease status.

Sex- and species-based comparisons (bottlenose dolphin vs. Pacific white-sided dolphin) also showed no significant differences in plasma ANP concentrations. Indo-pacific bottlenose dolphins and the hybrid dolphin were excluded from interspecies analyses due to the limited sample size.

**Figure 6:**
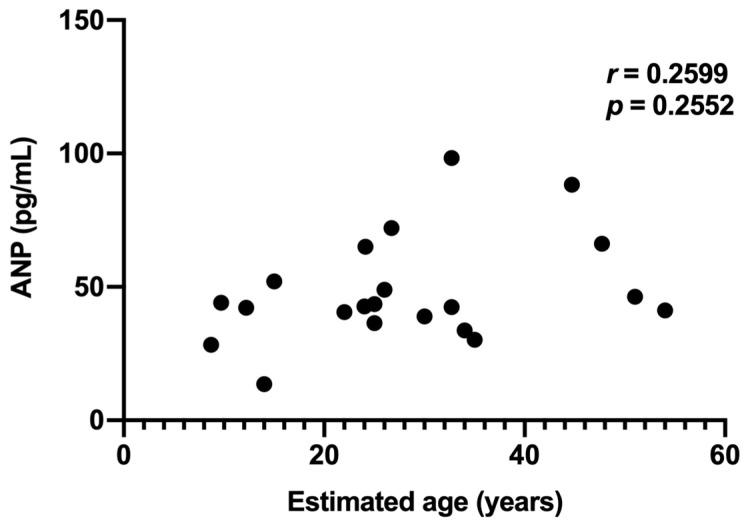
Scatter plot showing the relationship between plasma ANP concentration and estimated age (*n*= 21). No significant correlation was observed.

In 21 dolphins from the young and aged groups, Spearman’s rank correlation between estimated age and plasma ANP concentration was weak and not statistically significant (r = 0.2599, *p* = 0.2552; Figure 6).

#### 3.2.2. Plasma BNP Levels and Detectability

Plasma BNP concentrations were below the detection threshold (2.0 pg/mL) in all samples.

## 4. Discussion

Transthoracic echocardiography in bottlenose dolphins and Pacific white-sided dolphins presents substantial technical challenges. Interference from lung tissue and the sternum consistently limited image quality, preventing detailed morphological and functional cardiac assessment. In addition, some individuals exhibited sensitivity to ultrasound equipment, indicating potential physiological and management burdens associated with the procedure. Given that some facilities may lack permanent echocardiographic equipment and that husbandry training levels vary, transthoracic echocardiography may be limited as a routine screening method in captive cetaceans.

Transesophageal echocardiography has been reported in restrained, non-sedated dolphins [3]; however, its utility may be limited by lung interference and the technical challenges associated with probe placement [22], restricting its application to selected individuals.

Kinoshita et al. (2024) [15] reported plasma ANP reference values in female bottlenose dolphins from a single facility. The present study expands upon this work by including multiple cetacean species and evaluating potential effects of age, sex, and mild disease. In addition, this study demonstrates the applicability of human-based ANP assay kits across these species.

**Figure 7:**
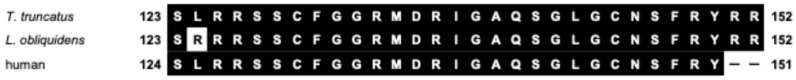
Alignment of ANP sequences in bottlenose dolphins (*Tursiops truncatus*), Pacific white-sided dolphins (*Lagenorhynchus obliquidens*), and humans.

The assay used targets the cyclic structure and C-terminal region of the ANP. The mature ANP forms a ring between the 7th and 23rd cysteines residues [7], and available data suggest that bottlenose dolphins and Pacific white-sided dolphins possess sequences homologous to humans [21]. This cross-reactivity indicates that commercially available human reagents can be applied in these species (Figure 7), which is particularly relevant given that their prevalence in managed care settings.

Although ANP sequences in Indo-pacific bottlenose dolphins and hybrid dolphins have not been fully characterized, successful detection suggests potential sequence homology. In contrast, BNP concentration was below the detection threshold in all samples, possibly reflecting greater interspecies divergence in BNP compared with ANP [8]. The absence of detectable BNP concentration in this study warrants further consideration. In humans and many domestic animals, BNP is widely used as a biomarker for cardiac disease, particularly in the diagnosis and monitoring of heart failure. However, the lack of detectable BNP in the present study may reflect several factors, including species-specific differences in peptide structure, gene expression, or secretion patterns [8,16]. It is also possible that the assay used in this study did not exhibit sufficient cross-reactivity with cetacean BNP, highlighting the importance of species-specific assay validation. These findings suggest that BNP may not be directly applicable as a cardiac biomarker in cetaceans without further molecular and analytical validation.

In terrestrial mammals such as humans and dogs, ANP is released in response to increased atrial wall tension and plays an important role in maintaining cardiovascular homeostasis [6,9]. ANP concentrations are known to increase in conditions associated with volume overload or cardiac dysfunction, reflecting its sensitivity to changes in cardiac preload [5,9,11]. The detection of measurable ANP concentrations across all individuals in the present study suggests that similar physiological mechanisms may be conserved in cetaceans. However, given the unique cardiovascular adaptations of cetaceans, including diving-related changes in blood distribution and pressure regulation, the regulation and interpretation of ANP dynamics may differ from those observed in terrestrial species [8]. These species-specific physiological differences should be carefully considered when interpreting ANP concentrations in cetaceans.

The reference range identified in this study (44.12 ± 14.62 pg/mL) is consistent with previous findings [15], supporting the robustness of ANP measurements across different populations. No significant effects of age, sex, species, or mild disease were observed; however, these findings should be interpreted with caution given the limited sample size. Although variation in ANP concentrations has been reported in adult dolphins [15], as well as age-related increases in humans [23], this trend was not evident in the present study. This may suggest a lack of age-related variation in this cohort; however, this observation should also be interpreted cautiously. This discrepancy may be related to differences in sample handling, such as temperature-dependent peptide degradation [24], or stress-related variability [25,26]. Species-specific physiological differences may also contribute.

In humans, plasma ANP concentrations may increase in non-cardiac conditions such as liver cirrhosis and hyperthyroidism [13,14]. In the present study, disease severity was not assessed in detail, which may have limited the detection of disease-related changes. Future studies including well-characterized cases are needed to clarify these relationships.

Cardiovascular abnormalities have been documented in stranded cetaceans, including valvular disease, myocardial infarction, and cardiomyopathy identified during necropsy [18,22,27]. Congenital cardiac anomalies have also been reported in neonatal dolphins [28,29]. These findings suggest that cardiovascular disease may be underdiagnosed in captive populations, particularly given their extended lifespan compared to wild counterparts [16].

The interpretation of cardiovascular biomarkers in cetaceans should also be considered in the context of their unique physiological adaptations to aquatic life. Diving behavior is associated with complex cardiovascular adjustments, including bradycardia, peripheral vasoconstriction, and redistribution of blood flow to essential organs [1]. These dynamic changes in hemodynamics may influence cardiac loading conditions and, consequently, the secretion of natriuretic peptides such as ANP. In addition, repeated diving and surfacing cycles may result in fluctuations in intrathoracic pressure and venous return, which could further modulate atrial stretch and peptide release. Furthermore, cetaceans exhibit species-specific differences in body size, metabolic rate, and activity patterns, all of which may contribute to variability in cardiovascular function [3,4]. For example, larger-bodied species may experience different baseline hemodynamic conditions compared to smaller species, potentially influencing circulating ANP concentrations. Similarly, variations in activity level, including training intensity and environmental enrichment, may affect cardiovascular demand and hormonal responses. These factors highlight the importance of considering both physiological and environmental context when interpreting biomarker data in managed cetacean populations.

Another important consideration is the potential influence of stress on ANP secretion. Although blood sampling in this study was performed voluntarily during routine husbandry procedures, individual variability in stress response cannot be completely excluded. Stress-related activation of neuroendocrine pathways may indirectly influence cardiovascular function and peptide release, introducing additional variability into measured concentrations [25,26]. This factor may be particularly relevant in zoological settings, where individual differences in temperament and training history can affect physiological responses to handling and environmental stimuli.

Taken together, these considerations suggest that ANP concentrations in cetaceans are likely influenced by a complex interplay of physiological, environmental, and management-related factors. As such, careful standardization of sampling conditions and detailed documentation of individual and facility-level variables will be important in future studies. A more comprehensive understanding of these influences will be essential for accurately interpreting ANP data and for assessing its potential role in health monitoring and research applications.

This study demonstrates that plasma ANP measurement using human reagents is feasible and may represent a promising approach for non-invasive assessment; however, its role as a cardiovascular screening tool requires further validation. In addition, accurate interpretation, particularly in less-studied species, requires further molecular characterization of peptide sequences. The absence of clear factors influencing ANP variability highlights the need for additional studies, especially in animals with confirmed cardiac or systemic disease.

These findings support the potential utility of ANP as a health-monitoring tool in captive marine mammals. Cetaceans are widely recognized sentinel species for both ocean and animal health [17], and the ability to assess physiological status using non-invasive biomarkers such as ANP may enhance their role in integrated health monitoring. Because ANP can be measured without sedation or invasive procedures, it may be particularly useful in zoological and aquarium settings where access to imaging modalities and implementation conditions may vary. However, a direct relationship between ANP concentration and objective indicators of cardiovascular disease was not established in this study, which limits the current clinical interpretation. Establishing species-specific baseline values could further contribute to long-term health management, especially in aging or high-risk individuals.

This study has several limitations, including a relatively small sample size, which may limit statistical power and suggests that the present findings should be interpreted as exploratory rather than confirmatory. Sample sizes for the Indo-pacific bottlenose dolphins (*n* = 2) and the hybrid dolphin (*n* = 1) were small, limiting generalizability. In addition, ANP sequences for these species have not been fully characterized, raising questions about assay specificity. This represents a major limitation when interpreting assay specificity and cross-reactivity. Although animals were classified as clinically healthy based on physical examination and available clinical assessments, the presence of subclinical or undetected disease cannot be completely excluded. Importantly, ANP concentrations were not evaluated in animals with cardiac or systemic disease, and therefore the relevance of these findings to diseased populations remains unknown and warrants dedicated investigation in clinically affected individuals. Environmental and institutional variations, including differences in husbandry practices, diet, and management conditions across facilities, may have influenced the measured ANP concentrations and should be considered potential confounding factors. In addition, the cross-sectional design of this study limits the ability to assess temporal changes or predictive value, highlighting the need for longitudinal studies. Future studies with larger, genetically defined populations and molecular validation are needed. Molecular verification of ANP sequences is essential before establishing reliable reference values.

In addition to the factors discussed above, variability in ANP concentrations may also be influenced by individual-level characteristics that were not fully captured in this study. For example, differences in body condition, hydration status, and metabolic state may affect cardiovascular dynamics and hormone secretion [24,25,26]. Similarly, subtle variations in daily routines, including feeding schedules and activity patterns, could contribute to intra- and inter-individual variability. Although efforts were made to collect samples under consistent conditions, complete standardization was not feasible in the present study, reflecting the practical constraints of working with managed cetaceans.

These considerations highlight the importance of integrating physiological, environmental, and management-related information when interpreting biomarker data. Future studies incorporating more detailed phenotypic and environmental data, as well as repeated sampling within individuals, may help to reduce variability and improve the interpretability of ANP measurements. Such approaches will be particularly important for distinguishing normal physiological variation from potential indicators of disease.

In summary, this study provides baseline data on plasma ANP concentrations in multiple cetacean species and highlights its potential role in non-invasive health monitoring. Although the present findings indicate that ANP can be measured in cetaceans using existing clinical assay systems, its biological and clinical relevance remains to be fully elucidated. Establishing the normal range and variability of ANP concentrations across different species, age groups, and environmental conditions will be essential for interpreting future data. In addition, longitudinal studies will be necessary to determine whether ANP reflects physiological changes over time or responds to the development of disease. Such investigations may contribute to a better understanding of cardiovascular physiology in cetaceans and support the development of minimally invasive monitoring approaches in managed care settings. However, these applications should be considered preliminary, and further validation in clinically affected populations is required before any clinical interpretation can be made. Future research should focus on disease-specific reference intervals, molecular characterization of natriuretic peptides, and longitudinal evaluation of ANP dynamics.

## 5. Conclusions

In conclusion, plasma ANP may provide preliminary insights into physiological variation in captive cetaceans; however, its potential utility as a biomarker remains uncertain and requires further investigation. This study should be considered exploratory, and additional research with larger sample sizes and broader taxonomic representation is needed to better understand ANP dynamics and to evaluate its relevance in clinical and health assessment contexts. Further integrative approaches combining biomarker analysis with imaging and clinical data may enhance the interpretation of cardiovascular status in cetaceans. In addition, continued efforts toward molecular characterization and assay validation will be essential to improve the reliability and interpretability of natriuretic peptide measurements across different cetacean species.

## Figures and Tables

**Table 1:** Individual dolphin characteristics and plasma ANP concentrations. Data are presented as mean (range), as appropriate.

	*T. truncatus*	*T. aduncus*	Hybrid *	*L. obliquidens*
Number (***n***)	13	2	1	10
Estimated age (years)	24.1 [8.7~44.7]	52.5 [51~54]	35	26.85 [14~47.7]
Sex (male/female, %)	2/11 (15.4/84.6)	1/1 (50.0/50.0)	0/1 (0/100.0)	7/3 (70.0/30.0)
Weight (kg)	245 [167.5~356]	185.5 [182~189]	224	115.15 [92~126.5]
ANP (pg/mL)	43.6 [28.3~98.3]	43.75 [41.2~46.3]	29.9	35.05 [11.1~171.9]

* Hybrid of a female bottlenose dolphin (*T. truncatus*) and male Indo-Pacific bottlenose dolphin (*T. aduncus*).

**Table 2:** Group classification used for statistical analysis. Data are presented as mean [range], as appropriate.

	Young	Aged	Diseased
Number (***n***)	12	9	5
Estimated age (years)	23 [8.7~26.7]	35 [30~54]	27 [14.3~37]
Sex (male/female, %)	4/8 (33.3/66.7)	3/6 (33.3/66.7)	3/2 (60.0/40.0)
Species (B/P/O, %)	9/3/0 (75.0/25.0/0.0)	4/3/2 (44.4/33.3/22.2)	0/4/1 (0.0/80.0/20.0)
Weight (kg)	230.5 [114~292]	189 [100.2~356]	117 [92~224]
ANP (pg/mL)	43.15 [13.5~72]	42.4 [30.2~98.3]	29.9 [11.1~171.9]

B, bottlenose dolphin (*Tursiops truncatus*); P, pacific white-sided dolphins (*Lagenorhynchus obliquidens*); O, other, Indo-Pacific bottlenose dolphins (*T. aduncus*) and the hybrid.

## Data Availability

The original contributions presented in this study are included in the article. Further inquiries can be directed to the corresponding authors.

## Abbreviations

The following abbreviations are used in this manuscript:

ANP	Atrial Natriuretic Peptide
BNP	Brain Natriuretic Peptide
CLEIA	Chemical luminescence enzyme immunoassay
